# Employment 12 months after kidney transplantation: An in-depth bio-psycho-social analysis of the Swiss Transplant Cohort

**DOI:** 10.1371/journal.pone.0175161

**Published:** 2017-04-27

**Authors:** Brigitta Danuser, Amira Simcox, Regina Studer, Michael Koller, Pascal Wild, for the Psychosocial Interest Group, Swiss Transplant Cohort Study

**Affiliations:** 1 Institut Universitaire Romand de Santé au Travail (Institute for Work and Health), University of Lausanne and University of Geneva, Epalinges - Lausanne, Switzerland; 2 Basel Institute for Clinical Epidemiology and Biostatistics, University Hospital Basel, Basel, Switzerland; 3 Institut national de recherche et de sécurité (INRS), Vandoeuvre Les Nancy Cedex, France; University of Toledo, UNITED STATES

## Abstract

**Background:**

Return to work with or after a chronic disease is a dynamic process influenced by a variety of interactions between personal, work, societal and medical resources or constraints. The aim of this study was to identify predictors for employment 12 months after transplantation in kidney patients, applying a bio-psycho-social model.

**Methods:**

All kidney patients followed in the Swiss Transplant Cohort between May 2008 and December 2012, aged 18 to 65 were assessed before, 6 and 12 months after transplantation.

**Results:**

Of the 689 included patients, 56.2% worked 12 months post- transplantation compared to 58.9% pre-transplantation. Age, education, self-perceived health (6 months post- transplantation), pre- transplantation employment and receiving an organ from a living donor are significant predictors of employment post- transplantation. Moreover, while self-perceived health increased post- transplantation, depression score decreased only among those employed 12 months post- transplantation. Pre- transplantation employment status was the main predictor for post- transplantation employment (OR = 18.6) and was associated with sex, age, education, depression and duration of dialysis. An organ from a living donor (42.1%) was more frequent in younger patients, with higher education, no diabetes and shorter waiting time to surgery.

**Conclusion:**

Transplantation did not increase employment in end-stage kidney disease patients but helped maintaining employment. Pre-transplantation employment has been confirmed to be the most important predictor of post-transplantation employment. Furthermore, socio-demographic and individual factors predicted directly and indirectly the post-transplantation employment status. With living donor, an additional predictor linked to social factors and the medical procedure has been identified.

## Introduction

Kidney transplantation (Tx) is currently the treatment of choice for end-stage renal disease. Already in 1995, Meyer [1] clearly identified role and social performance as indicators of function status for the Medical Outcome Studies. Employment plays a key role in social participation in the productive years of a person. For kidney patients, employment significantly contributes to their general well-being, mental health and quality of life [2]. In order to support Tx patients in returning back to work, a deeper understanding of the predictors of this process is crucial.

Return to work with or after a chronic disease is a dynamic process influenced by a variety of interactions between personal resources or constraints (e.g. age, functional capacity, education, health perception, mental health), work and working conditions (physical demands, psychosocial factors, income) and societal and medical factors (welfare system, health care access, treatment demands) [3]. Such a process has to be analyzed with a broad bio-psycho-social model [3, 4].

Employment rates after kidney Tx vary widely from as low as 28% to as high as 58% [4–8]. Over the past years, several predictors of not being employed post-Tx in kidney patients have been identified: Post-Tx employment status was consistently and highly correlated with pre-Tx employment status [4–7, 9–11]. Receiving a kidney from a living donor was regularly found to enhance social participation [12] and to specifically enhance the chance to be employed [6, 9, 13, 14]. Transplanted populations in general represent an aging population and most of these patients have a long disease history. When approaching the age of around 58 years, patients with a chronic disease in Switzerland often qualify for either invalidity pension or preretirement agreements [15]. Most studies found that being younger is a strong predictor of being employed post-Tx. The same is true for education. The higher the education level the more likely the transplanted patients will be employed post-Tx [5, 6, 9, 13, 16]. The influence of sex is contradictory: previous studies either reported a negative influence on post-Tx employment [5, 6] or no influence [9, 16].

With respect to bio-medical factors, several studies found that diabetes as cause for Tx is negatively associated with employment post-Tx [5, 13, 17] whereas Messias [14] and Markell [7] did not find any such relationship. Indicators for complications [16], post-operative complications [6], comorbidity [12], and blood pressure [4] showed no influence on employment post-Tx. Findings with respect to the influence of creatinine on employment post-Tx are mixed [4, 12]. In recent studies, a negative influence of the duration of dialyses pre-Tx and a positive influence of pre-emptive Tx on employment have been reported [6, 9].

Also quality of life factors such as physical or mental health indicators have been linked to post-Tx employment status in some cross-sectional or retrospective studies [5, 7, 16, 18]. However, these studies are susceptible to bias due to their design, especially with respect to subjective indicators. Thus, the results are inconclusive. It is, therefore, essential to test these factors in a prospective cohort. To our knowledge, the only prospective cohort study analyzing predictors of post-Tx employment including the pre-Tx, Tx, and post-Tx periods has been published in 1996 [13]. In the present study, we analyzed the potential influencing factors in a representative sample of kidney patients by linking medical data and psycho-social questionnaire data at study inclusion and follow-up.

The goal of this study was threefold:

1. To confirm the influence of previously identified predictors of post-Tx employment status: age, sex, socioeconomic status (income and education), pre-Tx employment status and living donor;
2. To study further the influence of: diabetes, waiting time, rejection, self-perceived health (SPH), and depression on post-Tx employment;
3. To analyze further the factors associated with the two potentially modifiable main predictors, which are hypothesized to be pre-Tx employment status and receiving a kidney from a living donor.

## Population and methods

### Study population

The study population was taken from the Swiss Transplant Cohort Study (STCS), a prospective multicenter cohort study including all organ recipients transplanted in Switzerland. All applicants for an organ transplant in Switzerland are included in the STCS when their application is registered and are followed up at 6 months, 12 months and at each subsequent year post-Tx including a psycho-social questionnaire and medically assessed data at each of the follow-up time-points. Interested readers are referred to De Geest et al. [19] and Koller et al. [20] for a more detailed description of the STCS. The research project was approved by the STCS scientific and ethical committee. All patients who did not give written informed consent for the use of their data were excluded from analysis.

For the present study, we selected all kidney patients recruited in the STCS database from May 2008 to December 2012, aged 18 to 65 at registration (which corresponds to the age interval between career entry and retirement in Switzerland). Excluded were double or previous transplants. Applying these criteria, 1 068 eligible patients were identified. Of these, employment status (main variable of interest) was missing pre-Tx for 242 patients and for 306 patients one year post-Tx. In total, the main outcome variable was missing for one or both assessment time points for 379 patients (35%) who were, thus, excluded from analysis. The final number for our study was 689 patients.

### Analyses

The main variable of interest, post-Tx employment status assessed 12 months post-Tx, is modeled as a function of sex, age, income, education and pre-Tx employment (all assessed at baseline), SPH and depression assessed 6 months post-Tx, the existence of a rejection episode during the 12 months following Tx, time on the waiting list (waiting time), donor type (living vs. deceased) assessed at Tx (Analysis I).

The two major predictors of post-Tx employment (identified both in the literature and in our data set), i.e., being employed pre-Tx and living donor were analyzed in more detail in a second stage: Pre-Tx employment status was investigated as a function of sex, age, income, education, SPH pre-Tx, depression pre-Tx, diabetes, and time since first dialysis assessed at inclusion in the cohort study (Analysis II).

Donor type (living vs. deceased) was modeled as a function of the same variables as mentioned above with the exception of time since first dialysis. Additionally, the pre-Tx employment status and waiting time was integrated as predictor variable (Analysis III).

### Variables

Pre-Tx employment status was defined as being employed, i.e., actively working in the labor market in a paid position (independent of the workload) or not employed, i.e., not being active in the paid labor market at the time of inclusion in the cohort based on self-reports. Since the focus of this study was on paid work, housewives/-men and students were considered as not employed. Post-Tx employment status was defined identically but referred to the time 12 months after kidney Tx.

Age (18–35, 35–45, 45–55, 55–65) and the current monthly income of the household after tax deductions were considered in 4 categories (< 4 500 CHF, 4 500–6 000 CHF, 6 001–9 000 CHF, > 9 000 CHF). Education was assessed in three categories (no professional education [no finished school or nine years of mandatory school]), professional education [apprenticeship, diploma qualifying for admission to university, mastery level diploma and federal diploma], higher professional education [higher technical or commercial school and University degree]). SPH was assessed with a visual analog scale at the time of filling out the psycho-social questionnaire (0 being the worst state imaginable and 100 being the best state) and considered in two categories with a cut-off point at 75. Depression was based on the HADS [21]. For the purpose of this study we only used the depression subscale consisting of seven items to be answered on a scale ranging from 0 to 3 yielding a total score from 0 to 21 for which we used a cut-off point at 7.

The following variables have been taken from the medical database: rejection (yes, no), waiting time until Tx (< 1.5 years; 1.6–4.5 years), diabetes (yes, no), living donor (yes, no). Waiting time until Tx represents the time since inclusion in the cohort study until Tx. Finally, time since first dialysis at inclusion was considered in three categories: none or less than one year, from 1 to 3 years, and more than three years.

### Statistical methods

The three analyses were based on logistic regression models. In a first stage, all independent variables were assessed each at a time in a simple regression. In a second stage, the statistically significant variables were included jointly in a multiple regression analysis and a backward selection procedure was applied in order to select a parsimonious model by dropping sequentially all non-significant variables from the model. Furthermore, all first order interactions in the three different regressions were analyzed. Given that the level of education and income were highly correlated, we considered only level of education in the multiple regression models as the number of missing values for income was much higher. All statistical significances were considered at a 5% level.

## Results

### Employment status 12 months post-Tx (Analysis l)

Of the 689 patients, 387 (56.2%) were employed 12 months after kidney Tx compared to 58.9% who worked pre-Tx. The main reason for not working were illness (31.1%), invalidity pension (26.5%), pre-retirement (20.9%), housewife/-man (13.9%), other reason (3.3%), unemployed (2%), missing (2%), and in education (0.3%).

Table 1 shows the descriptive results of the considered predictor variables of being employed and not employed 12 months post-Tx in kidney patients, as well as the simple and the multiple regression models. In the multiple regression model, being younger, having higher professional education, having better SPH 6 month post-Tx, receiving a kidney from a living donor and being employed pre-Tx were shown to significantly contribute to employment 12 months post-Tx. Waiting time and diabetes were only significant in the simple regression. Sex and rejection did not even reach statistical significance in the simple regression analysis.

**Table 1:** Predictors of employment status 12 months post-Tx in all kidney patients (descriptive results and logistic regression analysis).

		Study population (N = 689)			
Predictors		Not employed post-Tx (12 months) N = 302 (43.8%)	Employed post-Tx (12 months) N = 387 (56.2%)	Simple regression OR [95% CI]; p value	Multiple regression N = 557 OR [95% CI]; p value
**Sex** (m = 0)					
	**Female** (n = 242–35%)	114 (47.1%)	128 (52.9%)	Reference	
	**Male** (n = 447–65%)	188 (42.1%)	259 (57.9%)	1.2 [0.9–1.7]; 0.203	
**Age class** (m = 3)					
	55–65 yrs (n = 238–35%)	144 (60.5%)	94 (39.5%)	Reference	Reference
	45–55 yrs (n = 200–29%)	82 (41.0%)	118 (59.0%)	2.2 [1.5–3.2]; 0.000	4.3 [2.3–8.1]; 0.000
	35–45 yrs (n = 146–21%)	44 (30.1%)	102 (68.9%)	3.6 [2.3–5.5]; 0.000	3.0 [1.5–5.8]; 0.001
	18–35 yrs (n = 102–15%)	31 (30.4%)	71 (69.6%)	3.5 [2.1–5.8]; 0.000	5.6 [2.5–12.4]; 0.000
**Income** (m = 114)					
	< 4 500 CHF (n = 247–43%)	141 (57.1%)	106 (42.9%)	Reference	
	4 500–6 000 CHF (n = 142–25%)	56 (39.4%)	86 (60.6%)	2.0 [1.3–3.1]; 0.001	
	6 001–9 000 CHF (n = 106–18%)	30 (28.3%)	76 (71.7%)	3.4 [2.1–5.5]; 0.000	
	> 9 000 CHF (n = 80–14%)	17 (21.3%)	63 (78.7%)	4.9 [2.7–8.9]; 0.000	
**Education** (m = 21)					
	No prof. education (n = 210–31%)	125 (59.5%)	85 (40.5%)	Reference	Reference
	Prof. education (n = 265–40%)	107 (40.4%)	158 (59.6%)	2.2 [1.5–3.1]; 0.000	1.6 [0.9–2.9]; 0.095
	Higher prof. Education (n = 193–29%)	62 (32.1%)	131 (67.9%)	3.1 [2.1–4.7]; 0.000	2.5 [1.3–4.7]; 0.003
**SPH 6 months post-Tx** (m = 71)					
	VAS score 0–75 (n = 306–50%)	175 (57.2%)	131 (42.8%)	Reference	Reference
	VAS score 75–100 (n = 312–50%)	90 (28.9%)	222 (71.2%)	3.2 [2.2–4.4]; 0.000	2.4 [1.4–4.0]; 0.001
**Depression 6 months post-Tx** (m = 101)					
	HADS score 8–21 (n = 76–13%)	50 (65.8%)	26 (34.2%)	Reference	Reference
	HADS score 0–7 (n = 512–87%)	201 (39.1%)	311 (60.7%)	3.1 [1.8–5.1]; 0.000	1.8 [0.8–3.7]; 0.104
**Rejection** (m = 0)					
	Yes (n = 217–31%)	95 (43.8%)	122 (56.2%)	Reference	
	No (n = 472–69%)	207 (43.9%)	265 (56.1%)	1.0 [0.7–1.4]; 0.985	
**Waiting time** (m = 3)					
	1.6–4.5 yrs (n = 124–18%)	69 (55.7%)	55 (44.4%)	Reference	
	< 1.5 yrs (n = 562–82%)	232 (41.3%)	330 (58.7%)	1.8 [1.2–2.6]; 0.004	
**Diabetes** (m = 0)					
	Yes (n = 89–13%)	52 (58.4%)	37 (41.6%)	Reference	
	No (n = 600–87%)	250 (41.7%)	350 (58.3%)	0.5 [0.3–0.8]; 0.003	
**Living donor** (m = 0)					
	No (n = 399–58%)	218 (54.6%)	181 (45.4%)	Reference	Reference
	Yes (n = 290–42%)	84 (29.0%)	206 (71.0%)	3.0 [2.1–4.1]; 0.000	2.8 [1.7–4.7]; 0.000
**Employment pre-Tx** (m = 0)					
	No (n = 283–58%)	228 (80.6%)	55 (19.4%)	Reference	Reference
	Yes (n = 406–42%)	74 (18.2%)	332 (81.8%)	18.6 [12.6–27.4]; 0.000	26.6 [15.4–47.7]; 0.000

Note: SPH = Self-perceived health; HADS = Hospital Anxiety Depression Scale; VAS = Visual Analog Scale; m = missing

Among all tested first order interactions, only the interaction between SPH 6 months post-Tx and age became significant (p = 0.04). In the youngest age class, SPH had a stronger effect on employment than in the older age classes.

### Pre-Tx employment status (Analysis ll)

As pre-Tx employment status was shown to be the most important predictor (OR = 26.6) for employment 12 months after kidney Tx in Analysis I, Table 2 shows the influencing factors of pre-Tx employment status. In the simple regression analysis, being a male, being between 35–45 years old, having an income higher than 4 500 CHF, having a higher education, having a higher SPH score and a lower depression score pre-Tx, and time since first dialysis less than a year are factors associated with patients more likely to be employed than not employed pre-Tx. Sixty-two percent of the patients received hemodialysis (1% of them as homedialysis), 16% peritoneal dialysis, 17% none and 5% were missing. The type of dialysis was not associated with the pre-Tx employment status. In the multiple regression model, being male, being younger, having higher education, having lower depression scores, and having a shorter time since first dialysis were found significantly associated with being employed pre-Tx measured at study inclusion time. Among all tested first order interactions only the interaction between SPH and depression became significant (p = 0.02). More precisely, among patients with higher depression scores the chance to be employed pre-Tx increased with increasing SPH. This association was not found for patients with low depression scores.

**Table 2:** Predictors of pre-Tx employment status in kidney patients.

		Study population (N = 689)			
Predictors		Not employed N = 283 (41.1%)	Employed N = 406 (58.9%)	Simple regression OR [95% CI]; p value	Multiple regression N = 572 OR [95% CI]; p value
**Sex** (m = 0)					
	Female (n = 242–35%)	115 (47.5%)	127 (52.5%)	Reference	Reference
	Male (n = 447–65%)	168 (37.6%)	279 (62.4%)	1.5 [1.1–2.1]; 0.012	1.5 [1.0–2.2]; 0.031
**Age class** (m = 3)					
	55–65 yrs (n = 238–35%)	113 (47.5%)	125 (52.5%)	Reference	Reference
	45–55 yrs (n = 200–29%)	82 (41.0%)	118 (59.0%)	1.3 [0.9–1.9]; 0.174	1.5 [0.9–2.2]; 0.098
	35–45 yrs (n = 146–21%)	42 (28.8%)	104 (71.2%)	2.2 [1.4–3.5]; 0.000	2.2 [1.3–3.7]; 0.003
	18–35 yrs (n = 102–15%)	44 (43.1%)	58 (56.9%)	1.2 [0.8–1.9]; 0.462	1.3 [0.7–2.3]; 0.420
**Income** (m = 114)					
	< 4 500 CHF (n = 247–43%)	130 (52.6%)	117 (47.4%)	Reference	
	4 500–6 000 CHF (n = 142–25%)	47 (33.1%)	95 (66.9%)	2.2 [1.5–3.5]; 0.000	
	6 001–9 000 CHF (n = 106–18%)	24 (22.6%)	82 (77.4%)	3.8 [2.3–6.4]; 0.000	
	> 9 000 CHF (n = 80–14%)	21 (26.3%)	59 (73.7%)	3.1 [1.8–5.4]; 0.000	
**Education** (m = 21)					
	No prof. education (n = 210–31%)	117 (55.7%)	93 (44.3%)	Reference	Reference
	Prof. education (n = 265–40%)	95 (35.9%)	170 (64.2%)	2.3 [1.6–3.3]; 0.000	1.6 [1.1–2.5]; 0.027
	Higher prof. education (n = 193–29%)	64 (33.1%)	129 (66.8%)	2.5 [1.7–3.8]; 0.000	2.0 [1.3–3.2]; 0.000
**SPH pre-Tx** (m = 34)					
	VAS score 0–75 (n = 489–75%)	211 (43.2%)	278 (56.9%)	Reference	Reference
	VAS score 75–100 (n = 166–25%)	54 (32.5%)	112 (67.5%)	1.6 [1.1–2.3]; 0.016	1.5 [1.0–2.4]; 0.052
**Depression pre-Tx** (m = 57)					
	HADS score 8–21 (n = 117–19%)	71 (60.7%)	46 (39.3%)	Reference	Reference
	HADS score 0–7 (n = 515–81%)	182 (35.3%)	333 (64.7%)	2.8 [1.9–4.3]; 0.000	2.6 [1.6–4.1]; 0.000
**Diabetes** (m = 0)					
	Yes (n = 89–13%)	39 (43.8%)	50 (56.2%)	Reference	
	No (n = 600–87%)	244 (40.7%)	356 (59.3%)	1.1 [0.7–1.8]; 0.573	
**Time since first dialysis** (m = 23)					
	More than 3 yrs (n = 220–33%)	111 (50.5%)	109 (49.6%)	Reference	Reference
	Between 1 and 3 yrs (n = 208–31%)	92 (44.2%)	116 (55.8%)	1.3 [0.9–1.9]; 0.198	0.9 [0.6–1.4]; 0.759
	Less than a year or none (n = 238–36%)	73 (30.7%)	165 (69.3%)	2.3 [1.6-.3.4]; 0.000	1.7 [1.1–2.7]; 0.017

Note: SPH = Self-perceived health; HADS = Hospital Anxiety Depression Scale; VAS = Visual Analog Scale; m = missing

### Donor type: Living vs. deceased donor (Analysis lll)

Table 3 shows the simple and multiple regression analysis of receiving a kidney from a living versus a non-living donor. In the simple regression analysis, patients are more likely to receive a kidney from a living donor if they are younger, have a higher income and a higher education, have lower depression scores, have a shorter waiting time, have no diabetes and if they are employed pre-Tx. In the multiple regression model, variables that remained statistically significant were: being younger, having a higher education, having a shorter waiting time and having no diabetes.

**Table 3:** Predictors of receiving a kidney donation from a living vs. a non-living donor.

		Kidney Donor			
Predictors		Living N = 290 (42.1%)	Non-living N = 399 (57.9%)	Simple regression OR [95% CI]; p value	Multiple regression N = 611 OR [95% CI]; p value
**Sex** (m = 0)					
	Female (n = 242–35%)	101 (41.7%)	141 (58.3%)	Reference	
	Male (n = 447–65%)	189 (42.3%)	258 (57.7%)	1.0 [0.7–1.4]; 0.890	
**Age class** (m = 3)					
	55–65 yrs (n = 238–35%)	81 (34.0%)	157 (66.0%)	Reference	Reference
	45–55 yrs (n = 200–29%)	85 (42.5%)	115 (57.5%)	1.4 [1.0–2.1]; 0.069	1.5 [0.9–2.4]; 0.076
	35–45 yrs (n = 146–21%)	70 (48.0%)	76 (52.0%)	1.8 [1.2–2.7]; 0.007	1.7 [1.0–2.7]; 0.043
	18–35 yrs (n = 102–15%)	52 (51.0%)	50 [49.0%)	2.0 [1.3–3.2]; 0.004	1.9 [1.1–3.3]; 0.029
**Income** (m = 114)					
	< 4 500 CHF (n = 247–43%)	69 (27.9%)	178 (72.1%)	Reference	
	4 500–6 000 CHF (n = 142–25%)	67 (47.2%)	75 (52.8%)	2.3 [1.5–3.5]; 0.000	
	6 001–9 000 CHF (n = 106–18%)	55 (51.9%)	51 (48.1%)	2.8 [1.7–4.5]; 0.000	
	> 9 000 CHF (n = 80–14%)	55 (68.8%)	25 (31.3%)	5.7 [3.3–9.8]; 0.000	
**Education** (m = 21)					
	No prof. education (n = 210–31%)	61 (29.1%)	149 (70.9%)	Reference	Reference
	Prof. education (n = 265–40%)	115 (43.4%)	150 (56.6%)	1.9 [1.3–2.8]; 0.001	2.0 [1.2–3.1]; 0.003
	Higher prof. education (n = 193–29%)	107 (55.4%)	86 (44.6%)	3.0 [2.0–4.6]; 0.000	3.0 [1.8–4.8]; 0.000
**SPH pre-Tx** (m = 34)					
	VAS score 0–75 (n = 489–75%)	202 (41.3%)	287 (58.7%)	Reference	
	VAS score 75–100 (n = 166–25%)	70 (42.2%)	96 (57.8%)	1.0 [0.7–1.5]; 0.846	
**Depression pre-Tx** (m = 57)					
	HADS score 8–21 (n = 117–19%)	36 (30.8%)	81 (69.2%)	Reference	Reference
	HADS score 0–7 (n = 515–81%)	234 (45.4%)	281 (54.6%)	1.9 [1.2–2.9]; 0.004	0.7 [0.4–1.1]; 0.106
**Waiting time** (m = 3)					
	1.6–4.5 yrs (n = 124–18%)	11 (8.9%)	113 (91.1%)	Reference	Reference
	< 1.5 yrs (n = 562–82%)	277 (49.3%)	285 (50.7%)	10.0 [5.3–18.9]; 0.000	9.8 [5.0–19.1]; 0.000
**Diabetes** (m = 0)					
	Yes (n = 89–13%)	16 (18.0%)	73 (82.0%)	Reference	Reference
	No (n = 600–87%)	274 (45.7%)	326 (54.3%)	3.8 [2.2–6.7]; 0.000	4.1 [2.2–7.7]; 0.000
**Employment pre-Tx** (m = 0)					
	No (n = 283–41%)	98 (34.6%)	185 (65.4%)	Reference	Reference
	Yes (n = 406–59%)	192 (47.3%)	214 (52.7%)	1.7 [1.2–2.3]; 0.001	1.3 [0.9–1.9]; 0.200

Note: SPH = Self-perceived health; HADS = Hospital Anxiety Depression Scale; VAS = Visual Analog Scale; m = missing

Among all tested first order interactions only the interaction between diabetes and depression became significant (p = 0.01). More precisely, among those patients with low depression scores diabetes patients have lower chances to receive an organ from a living donor compared to patients without diabetes. In patients with high depression scores, no such difference could be observed.

### Self-perceived health and depressive symptoms

**Fig 1:**
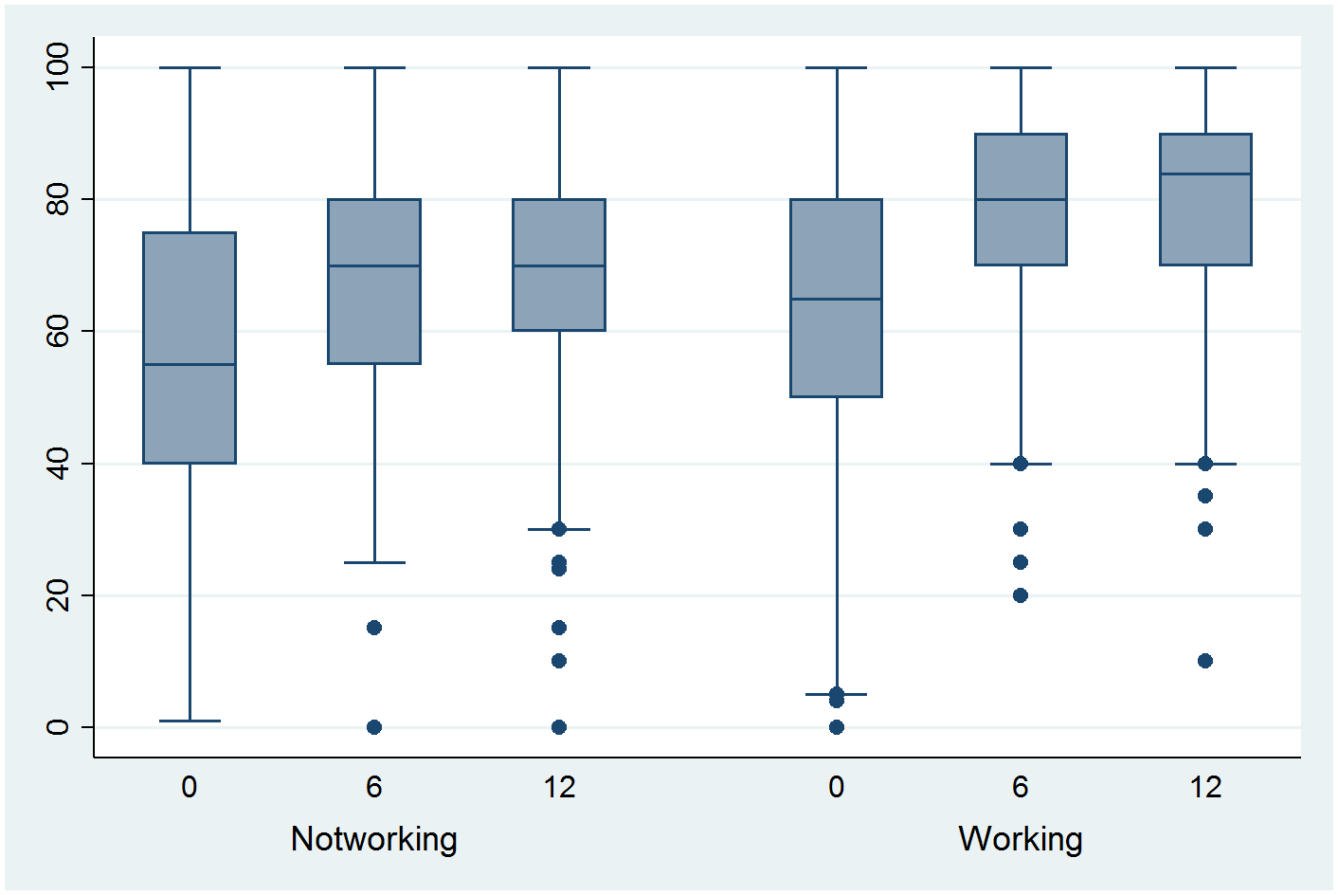
Evolution of self-perceived health (visual analogue scale) pre-Tx (0), 6 and 12 months post-Tx, by employment status 12 months post-Tx.

**Fig 2:**
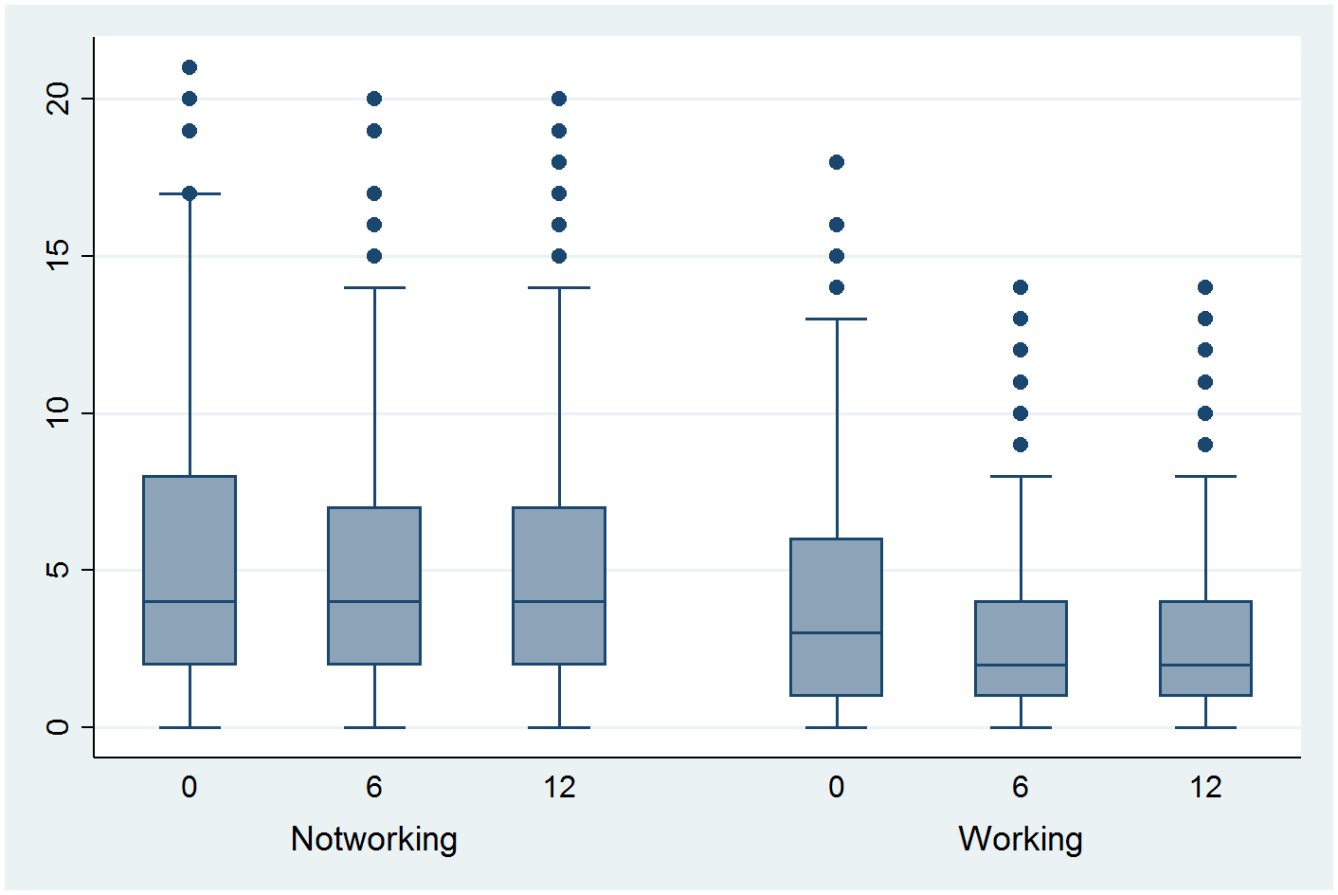
Evolution of depression pre-Tx (0), 6 and 12 months post-Tx, by employment status 12 months post-Tx.

As SPH 6 months post-Tx was found to be a predictor for employment status and as it is a potentially modifiable factor, the evolution of SPH (Fig 1) and of depression (Fig 2) based on the depression subscale of the Hospital Anxiety Depression Scale (HADS) are shown in Figs 1 and 2, respectively (separated by employment group, i.e., those patients who are working 12 months post-Tx and those who are not). SPH increased significantly 6 months post-Tx compared with pre-Tx independently of employment status (*p* < 0.0001). SPH pre-Tx and post-Tx were significantly different in the two groups (*p* < 0.0001). In both groups, SPH increased also significantly between 6 month and 12 months post-Tx. With respect to the HADS, the score decreased significantly between pre-Tx and 6 months post-Tx only in the group working 12 months post-Tx (*p* = 0.03).

## Discussion

Less persons are employed 12 months after kidney Tx than before despite a sharp increase in SPH post-Tx as shown in these analyses of the prospective national Swiss Transplant Cohort. Our results confirm that pre-Tx employment, living donor, younger age, higher education and, additionally, a better SPH 6 month post-Tx are predictors of 12 months post-Tx employment. Rejection problems and sex showed no influence on post-Tx employment.

56.2% kidney transplanted persons worked 12 months post-Tx. Comparable results were found in reports from the US [11] and from Belgium [22] with 56% and 58% employed post-Tx, respectively. Compared to other studies, the post-Tx employment rate is high [14, 16, 17, 23, 24]. This is mostly due to an already high employment rate of 58.9% pre-Tx in our cohort. This rate is considerably higher than in other European (Denmark 22% [25] Finland 30% [17] or US studies [26, 27]. As we measured pre-Tx employment status at inclusion time, our pre-Tx employment rate is probably slightly overestimated. When waiting time until Tx is long, some patients may have lost their employment in the meantime. This may represent a possible bias in our results. However, the waiting time was quite short (less than one year) in 82% of our population.

We found less kidney transplanted patients working post-Tx than before, a finding in agreement with Nour et al. [16] and Tzvetanov et al. [23], but in opposition to a recent Swiss study [9] that reported more kidney transplanted patients working post-Tx (71%) than before. The latter study is a retrospective monocentric survey with a response rate of 55% and may, therefore, have overestimated employment status due to a possible selection bias. The same authors also reported a very high pre-Tx employment rate of 65.7%.

### Pre-Tx employment

In agreement with most return to work studies taking pre-Tx employment status into account [4–7, 9–11], pre-Tx employment was the strongest predictor for post-Tx employment. 81% of the patients who were employed pre-Tx were employed 12 month post-Tx, versus 18% of those who were not employed pre-Tx. Sandhu et al. [27] showed in the US that having an employment gives a privileged access to Tx and shortens waiting time until Tx. If a society wishes to increase work participation post-Tx, the support to keep chronically ill persons at work has to start long before Tx and has to include return to work in the planning of Tx. The separate analyses of factors associated with pre-Tx employment status showed that being a male, being younger, having a higher education, higher SPH, less depressive symptoms and no dialyses or for less than one year are associated with being employed pre-Tx. The influence of a short dialyses period or pre-emptive Tx has recently emerged [6, 9, 10, 28]. Maintaining professional employment while being on long-term dialyses is hard to achieve due to the time-consuming treatment and its side-effects [25, 29]. The type of dialysis was not associated with the pre-Tx employment status.

### Living donor

We found that the percentage of employed patients post-Tx was higher amongst the living donor recipients than the non-living donor recipients (71% versus 45.4%). This result is in agreement with several recent studies [9, 13, 14, 30] but was not found by Nour et al. [16]. The rate of 42.1% of patients who organize a living donor is in the middle to upper field of reported results [9, 16, 30–34].

The recipients of a living donor in our study are younger, well educated and do not have diabetes as cause of their kidney pathology. Especially US studies showed sex, racial and socio-economic disparities in the utilization of living donor transplants [31, 35]. Our findings of a clear influence of age and education can be best interpreted with health literacy capacity in line with Dageforde et al. [36]. The finding that very few diabetes patients utilize a living donor was to our knowledge never described before. Further research is needed on this aspect.

It is well known that organs from a living donor show better functioning and a longer survival rate [37] but the differences are not massive [33] and are insufficient in explaining the influence of this factor on post-Tx employment. Receiving a living donor organ means a lot more than better organ functioning. It means searching actively for a donor and, in case the search is successful, it means actively and concretely planning the whole procedure instead of enduring an unknown waiting time with possible negative consequences for health [38]. Thus, the association of receiving a living donor organ with post-Tx employment might not be causal but might rather reflect, firstly, a general attitude of the organ receiver and, secondly, the benefits of a reduced and foreseeable waiting time for surgery. Additionally, other factors such as social network and family support that were not tested in this study may influence receiving an organ from a living donor. Our analyses of living and non-living donors show that receiving a living donor is highly correlated with a shorter waiting time until Tx, a variable showing an influence on post-Tx employment in the single regression and which was also found by Nour et al. [16] and Eng et al. [6] In line with this, Ismail et al. [32] found that living donor recipients spend less time on dialyses.

### Age, education, sex and SPH

Age was, as expected, an independent predictor of post-Tx employment [6, 9, 14, 17, 39] meaning that younger patients were more likely to be employed 12 months post-Tx. But age was also a strong mediator concerning being employed pre-Tx and receiving a living donor.

The level of education accomplished pre-Tx is a predictor for being employed post-Tx. More precisely, the higher the education, the higher the chance of being employed post-Tx. Positive relationship between employment status and education level is well known in kidney transplanted persons [5, 6]. Education had a direct influence on post-Tx employment, but like age, education is also a strong mediator for pre-Tx employment and for organizing a living donor. On the one hand, education gives access to better paid jobs with less physical demands, which are more adequate to the physical fitness of transplant recipients. On the other hand, education is a key factor concerning health literacy. Gender was only associated with pre-Tx employment status, but not with post-Tx employment or receiving a living donor. The male / female ratio of pre-Tx and post-Tx employment status is the same, i.e. 1.23, and corresponds to the Swiss labor market participation for women and men (2 414 000 male / 2 020 000 female = 1,22) [15] indicating a societal phenomenon with fewer women employed but rather working in the household.

Overall, Tx increased SPH significantly, whereas depression scores decreased significantly only in those patients who were employed post-Tx. Most studies researching quality of life aspects have focused on the post-Tx period and observed lower quality of life aspects in the mental and, particularly, in the physical domain compared to the general population [18, 40–42]. Only a French follow-up study post-Tx by Villeneuve et al. [43] showed that the physical and mental domain factors increased over the first months to achieve a level comparable with the general population around 6 months post-Tx. Our data give a similar picture for SPH and show even a further increase in SPH between 6 and 12 months. Additionally, we show that the group employed 12 months post-Tx has at each assessment point a higher SPH than the non-working group. The SPH values of the group working 12 months post-Tx (mean 80.4) are similar to those of 800 town employees in Switzerland aged 45–65 years (mean 79.1) [44]. Also others showed that those employed post-Tx have a better quality of life especially in the physical domain [11, 28, 38]. Additionally, a high SPH 6 month post-Tx predicted being employed 12 months post-Tx. This is in line with Sangalli et al. [11] who showed in a follow-up study post-Tx that good physical health perception enhances the chances of being employed.

Compared to other studies with chronically ill persons [45, 46], the HADS depression scores in our population are quite low and are comparable to the general population [47]. Depression 6 months post-Tx was significant in the single regression analyses with employment but not in the final model. This is in line with Saab et al. [48] who also found that there is no relation between mental health and employment status with the exception of liver patients. Contrary to the findings reported above, Gorevski et al. [49] and Bohlke et al. [5] found that post-Tx depression is associated with not being employed post-Tx. The conflicting results with respect to the association between depression and employment might be due to several reasons: no causal relationship in cross sectional studies, different methods to assess depression, and differences in time points between Tx and mental health assessment.

The employment rate post-Tx in this study has to be considered as suboptimal. Less persons are employed 12 months post-Tx than before despite a considerable increase in SPH. We found no clear reason for this phenomenon and restricting the analyses to kidney patients at younger age revealed the same results. The main reasons for not working post-Tx were illness, invalidity pension, and pre-retirement. Already Markell et al. [7] found that persons with an invalidity pension are less likely to be employed post-Tx. This was confirmed by recent studies [50]. In line with this, Sangalli et al. [11] and Tzvetanov et al. [23] reported lower employment rates post-Tx in persons insured by public insurances and Nour et al. [16] reported an increase in retirement rates from pre-Tx (8.3%) to post-Tx (18.3%). Another explanation may be that transplanted persons still feel handicapped and perceive their work-ability as insufficient despite the increase in health perception such as described by Slakey and Rosner [8]. These authors reported a striking contrast between the percentage of transplanted patients who were working (28%) and the percentage of those who were feeling able to work (60%).

We identified potentially modifiable factors which could be acted upon to improve work participation in kidney recipients. First, maintaining employment pre-Tx should be actively supported and, in case employment is lost, return to work and employability should be integrated in the Tx planning. Second, emphasis should be given to health perception. With this respect, physical rehabilitation might be particularly useful.

This study has several strengths: To our knowledge, this is the first study assessing quality of life factors in a prospective cohort and analyzing their influence on employment. The present study is a nation-wide multicenter study involving the six major hospitals in Switzerland so that we can assume a minimal selection bias. Contrary to other cohort studies with a limited number of patients, our study is based on a large and representative population.

One of the limitations of our study is the loss of approximately 35% of the initial population for analysis due to missing data concerning the main variable of interest, i.e., employment status. Other limitations are related to the questionnaire used, where detailed job characteristics (e.g., physically demanding jobs) were not assessed, and where there was no direct question on invalidity or retirement status.

## Conclusion

This study shows a suboptimal employment rate in end-stage kidney disease patients before and after Tx in a nationwide European cohort. The percentage of patients employed post-Tx was not higher but rather comparable to the percentage of patients employed pre-Tx. Thus, Tx did not increase post-Tx employment but it contributed to maintain employment in around 80% of the patients who were employed pre-Tx and allowed resuming work for around 20% of the patients not employed pre-Tx. Without Tx, a significant decrease in employment would have been expected due to the fact that the risk not to be employed increases with increasing time on dialysis.

With respect to the predictors of being employed one year post-Tx, this study confirms that being employed pre-Tx is the most influential one. Also socio-demographic factors such as age and education as well as SPH play an important direct but also mediating role on being employed post-Tx. Finally, receiving an organ from a living donor was identified as another predictor for being employed 12 months post-Tx.

## Abbreviations

HADS: Hospital Anxiety Depression Scale
SPH: Self-perceived Health
STCS: Swiss Transplant Cohort Study
Tx: Transplantation
VAS: Visual Analog Scale
